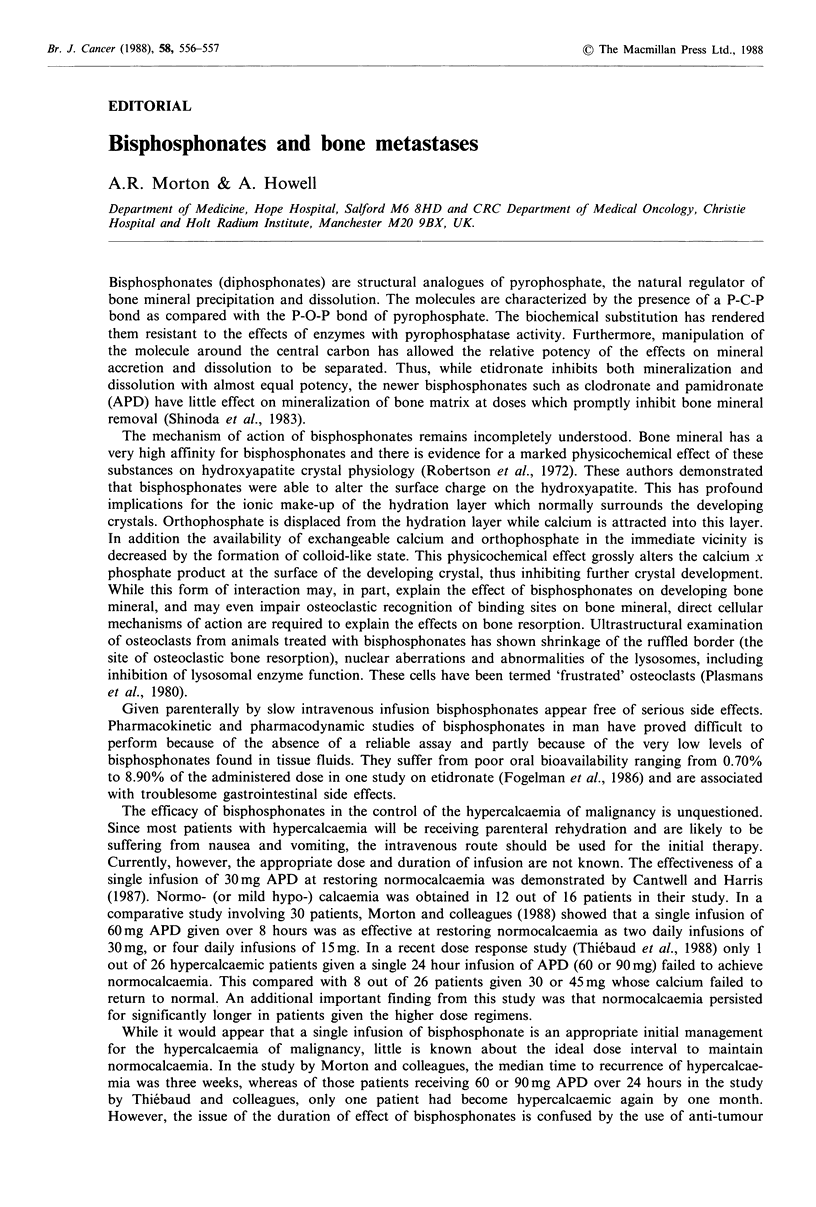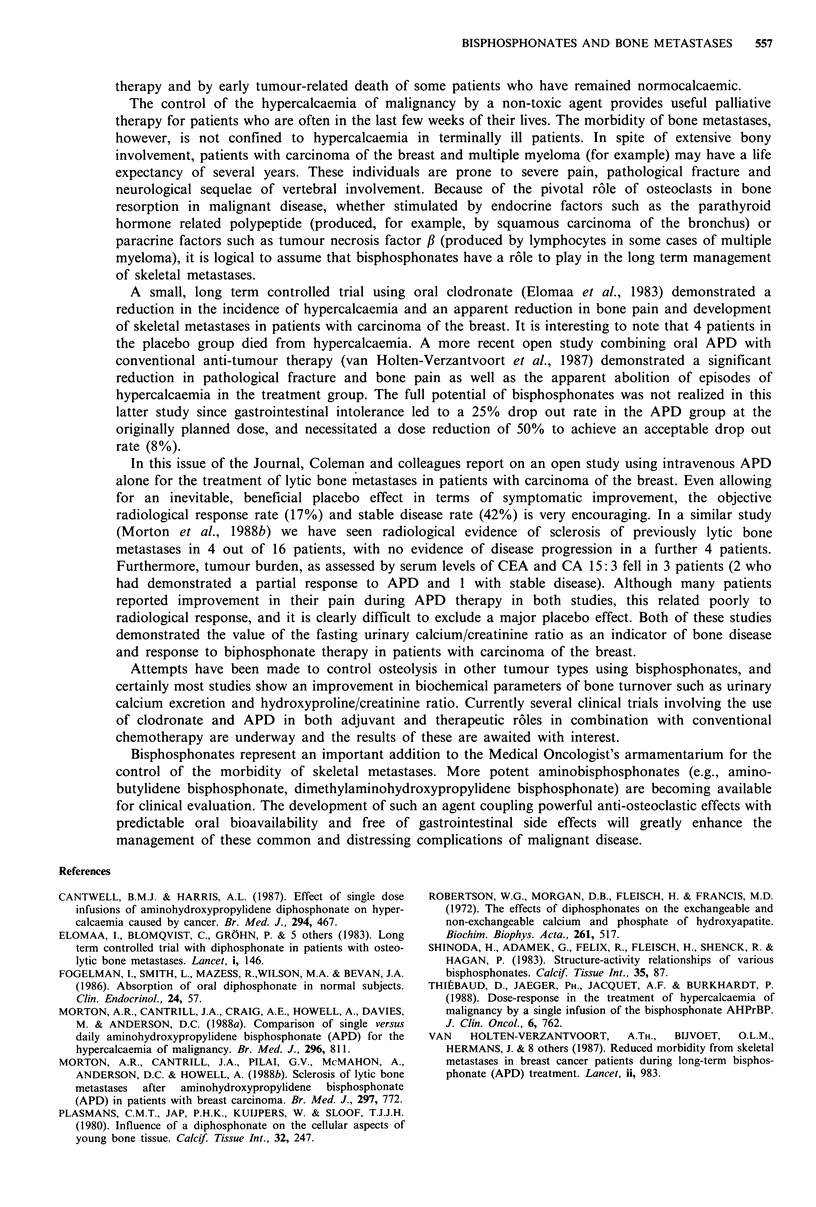# Bisphosphonates and bone metastases.

**DOI:** 10.1038/bjc.1988.257

**Published:** 1988-11

**Authors:** A. R. Morton, A. Howell

**Affiliations:** Department of Medicine, Hope Hospital, Salford, UK.


*Br. J. Cancer* (1988), **58**, 556–557 © The Macmillan Press Ltd., 1988

EDITORIAL

### Bisphosphonates and bone metastases

A.R. Morton & A. Howell

*Department of Medicine, Hope Hospital, Salford M6 8HD and CRC Department of Medical Oncology, Christie Hospital and Holt Radium Institute, Manchester M20 9BX, UK.*

Bisphosphonates (diphosphonates) are structural analogues of pyrophosphate, the natural regulator of bone mineral precipitation and dissolution. The molecules are characterized by the presence of a P-C-P bond as compared with the P-O-P bond of pyrophosphate. The biochemical substitution has rendered them resistant to the effects of enzymes with pyrophosphatase activity. Furthermore, manipulation of the molecule around the central carbon has allowed the relative potency of the effects on mineral accretion and dissolution to be separated. Thus, while etidronate inhibits both mineralization and dissolution with almost equal potency, the newer bisphosphonates such as clodronate and pamidronate (APD) have little effect on mineralization of bone matrix at doses which promptly inhibit bone mineral removal (Shinoda *et al.*, 1983).

The mechanism of action of bisphosphonates remains incompletely understood. Bone mineral has a very high affinity for bisphosphonates and there is evidence for a marked physicochemical effect of these substances on hydroxyapatite crystal physiology (Robertson *et al.*, 1972). These authors demonstrated that bisphosphonates were able to alter the surface charge on the hydroxyapatite. This has profound implications for the ionic make-up of the hydration layer which normally surrounds the developing crystals. Orthophosphate is displaced from the hydration layer while calcium is attracted into this layer. In addition the availability of exchangeable calcium and orthophosphate in the immediate vicinity is decreased by the formation of colloid-like state. This physicochemical effect grossly alters the calcium $x$ phosphate product at the surface of the developing crystal, thus inhibiting further crystal development. While this form of interaction may, in part, explain the effect of bisphosphonates on developing bone mineral, and may even impair osteoclastic recognition of binding sites on bone mineral, direct cellular mechanisms of action are required to explain the effects on bone resorption. Ultrastructural examination of osteoclasts from animals treated with bisphosphonates has shown shrinkage of the ruffled border (the site of osteoclastic bone resorption), nuclear aberrations and abnormalities of the lysosomes, including inhibition of lysosomal enzyme function. These cells have been termed 'frustrated' osteoclasts (Plasmans *et al.*, 1980).

Given parenterally by slow intravenous infusion bisphosphonates appear free of serious side effects. Pharmacokinetic and pharmacodynamic studies of bisphosphonates in man have proved difficult to perform because of the absence of a reliable assay and partly because of the very low levels of bisphosphonates found in tissue fluids. They suffer from poor oral bioavailability ranging from 0.70% to 8.90% of the administered dose in one study on etidronate (Fogelman *et al.*, 1986) and are associated with troublesome gastrointestinal side effects.

The efficacy of bisphosphonates in the control of the hypercalcaemia of malignancy is unquestioned. Since most patients with hypercalcaemia will be receiving parenteral rehydration and are likely to be suffering from nausea and vomiting, the intravenous route should be used for the initial therapy. Currently, however, the appropriate dose and duration of infusion are not known. The effectiveness of a single infusion of 30 mg APD at restoring normocalcaemia was demonstrated by Cantwell and Harris (1987). Normo- (or mild hypo-) calcaemia was obtained in 12 out of 16 patients in their study. In a comparative study involving 30 patients, Morton and colleagues (1988) showed that a single infusion of 60 mg APD given over 8 hours was as effective at restoring normocalcaemia as two daily infusions of 30 mg, or four daily infusions of 15 mg. In a recent dose response study (Thiébaud *et al.*, 1988) only 1 out of 26 hypercalcaemic patients given a single 24 hour infusion of APD (60 or 90 mg) failed to achieve normocalcaemia. This compared with 8 out of 26 patients given 30 or 45 mg whose calcium failed to return to normal. An additional important finding from this study was that normocalcaemia persisted for significantly longer in patients given the higher dose regimens.

While it would appear that a single infusion of bisphosphonate is an appropriate initial management for the hypercalcaemia of malignancy, little is known about the ideal dose interval to maintain normocalcaemia. In the study by Morton and colleagues, the median time to recurrence of hypercalcaemia was three weeks, whereas of those patients receiving 60 or 90 mg APD over 24 hours in the study by Thiébaud and colleagues, only one patient had become hypercalcaemic again by one month. However, the issue of the duration of effect of bisphosphonates is confused by the use of anti-tumour therapy and by early tumour-related death of some patients who have remained normocalcaemic.

The control of the hypercalcaemia of malignancy by a non-toxic agent provides useful palliative therapy for patients who are often in the last few weeks of their lives. The morbidity of bone metastases, however, is not confined to hypercalcaemia in terminally ill patients. In spite of extensive bony involvement, patients with carcinoma of the breast and multiple myeloma (for example) may have a life expectancy of several years. These individuals are prone to severe pain, pathological fracture and neurological sequelae of vertebral involvement. Because of the pivotal rôle of osteoclasts in bone resorption in malignant disease, whether stimulated by endocrine factors such as the parathyroid hormone related polypeptide (produced, for example, by squamous carcinoma of the bronchus) or paracrine factors such as tumour necrosis factor $\beta$ (produced by lymphocytes in some cases of multiple myeloma), it is logical to assume that bisphosphonates have a rôle to play in the long term management of skeletal metastases.

A small, long term controlled trial using oral clodronate (Elomaa *et al.*, 1983) demonstrated a reduction in the incidence of hypercalcaemia and an apparent reduction in bone pain and development of skeletal metastases in patients with carcinoma of the breast. It is interesting to note that 4 patients in the placebo group died from hypercalcaemia. A more recent open study combining oral APD with conventional anti-tumour therapy (van Holten-Verzantvoort *et al.*, 1987) demonstrated a significant reduction in pathological fracture and bone pain as well as the apparent abolition of episodes of hypercalcaemia in the treatment group. The full potential of bisphosphonates was not realized in this latter study since gastrointestinal intolerance led to a 25% drop out rate in the APD group at the originally planned dose, and necessitated a dose reduction of 50% to achieve an acceptable drop out rate (8%).

In this issue of the Journal, Coleman and colleagues report on an open study using intravenous APD alone for the treatment of lytic bone metastases in patients with carcinoma of the breast. Even allowing for an inevitable, beneficial placebo effect in terms of symptomatic improvement, the objective radiological response rate (17%) and stable disease rate (42%) is very encouraging. In a similar study (Morton *et al.*, 1988*b*) we have seen radiological evidence of sclerosis of previously lytic bone metastases in 4 out of 16 patients, with no evidence of disease progression in a further 4 patients. Furthermore, tumour burden, as assessed by serum levels of CEA and CA 15:3 fell in 3 patients (2 who had demonstrated a partial response to APD and 1 with stable disease). Although many patients reported improvement in their pain during APD therapy in both studies, this related poorly to radiological response, and it is clearly difficult to exclude a major placebo effect. Both of these studies demonstrated the value of the fasting urinary calcium/creatinine ratio as an indicator of bone disease and response to biphosphonate therapy in patients with carcinoma of the breast.

Attempts have been made to control osteolysis in other tumour types using bisphosphonates, and certainly most studies show an improvement in biochemical parameters of bone turnover such as urinary calcium excretion and hydroxyproline/creatinine ratio. Currently several clinical trials involving the use of clodronate and APD in both adjuvant and therapeutic rôles in combination with conventional chemotherapy are underway and the results of these are awaited with interest.

Bisphosphonates represent an important addition to the Medical Oncologist's armamentarium for the control of the morbidity of skeletal metastases. More potent aminobisphosphonates (e.g., amino-butylidene bisphosphonate, dimethylaminohydroxypropylidene bisphosphonate) are becoming available for clinical evaluation. The development of such an agent coupling powerful anti-osteoclastic effects with predictable oral bioavailability and free of gastrointestinal side effects will greatly enhance the management of these common and distressing complications of malignant disease.

### References

CANTWELL, B.M.J. & HARRIS, A.L. (1987). Effect of single dose infusions of aminohydroxypropylidene diphosphonate on hypercalcaemia caused by cancer. *Br. Med. J.*, **294**, 467.

ELOMAA, I., BLOMQVIST, C., GRÖHN, P. & 5 others (1983). Long term controlled trial with diphosphonate in patients with osteolytic bone metastases. *Lancet*, **i**, 146.

FOGELMAN, I., SMITH, L., MAZESS, R.,WILSON, M.A. & BEVAN, J.A. (1986). Absorption of oral diphosphonate in normal subjects. *Clin. Endocrinol.*, **24**, 57.

MORTON, A.R., CANTRILL, J.A., CRAIG, A.E., HOWELL, A., DAVIES, M. & ANDERSON, D.C. (1988*a*). Comparison of single *versus* daily aminohydroxypropylidene bisphosphonate (APD) for the hypercalcaemia of malignancy. *Br. Med. J.*, **296**, 811.

MORTON, A.R., CANTRILL, J.A., PILAI, G.V., McMAHON, A., ANDERSON, D.C. & HOWELL, A. (1988*b*). Sclerosis of lytic bone metastases after aminohydroxypropylidene bisphosphonate (APD) in patients with breast carcinoma. *Br. Med. J.*, **297**, 772.

PLASMANS, C.M.T., JAP, P.H.K., KUIJPERS, W. & SLOOF, T.J.J.H. (1980). Influence of a diphosphonate on the cellular aspects of young bone tissue. *Calcif. Tissue Int.*, **32**, 247.

ROBERTSON, W.G., MORGAN, D.B., FLEISCH, H. & FRANCIS, M.D. (1972). The effects of diphosphonates on the exchangeable and non-exchangeable calcium and phosphate of hydroxyapatite. *Biochim. Biophys. Acta.*, **261**, 517.

SHINODA, H., ADAMEK, G., FELIX, R., FLEISCH, H., SHENCK, R. & HAGAN, P. (1983). Structure-activity relationships of various bisphosphonates. *Calcif. Tissue Int.*, **35**, 87.

THIÉBAUD, D., JAEGER, PH., JACQUET, A.F. & BURKHARDT, P. (1988). Dose-response in the treatment of hypercalcaemia of malignancy by a single infusion of the bisphosphonate AHPrBP. *J. Clin. Oncol.*, **6**, 762.

VAN HOLTEN-VERZANTVOORT, A.TH., BIJVOET, O.L.M., HERMANS, J. & 8 others (1987). Reduced morbidity from skeletal metastases in breast cancer patients during long-term bisphosphonate (APD) treatment. *Lancet*, **ii**, 983.